# Shelf–ocean exchange and hydrography west of the Antarctic Peninsula: a review

**DOI:** 10.1098/rsta.2017.0164

**Published:** 2018-05-14

**Authors:** C. Moffat, M. Meredith

**Affiliations:** 1 School of Marine Science and Policy, University of Delaware, Newark, DE 19716, USA; 2 British Antarctic Survey High Cross, Madingley Road, Cambridge CB3 0ET, UK

**Keywords:** ocean, Antarctica, West Antarctic Peninsula

## Abstract

The West Antarctic Peninsula (WAP) is a highly productive marine ecosystem where extended periods of change have been observed in the form of glacier retreat, reduction of sea-ice cover and shifts in marine populations, among others. The physical environment on the shelf is known to be strongly influenced by the Antarctic Circumpolar Current flowing along the shelf slope and carrying warm, nutrient-rich water, by cold waters flooding into the northern Bransfield Strait from the Weddell Sea, by an extensive network of glaciers and ice shelves, and by strong seasonal to inter-annual variability in sea-ice formation and air–sea interactions, with significant modulation by climate modes like El Niño–Southern Oscillation and the Southern Annular Mode. However, significant gaps have remained in understanding the exchange processes between the open ocean and the shelf, the pathways and fate of oceanic water intrusions, the shelf heat and salt budgets, and the long-term evolution of the shelf properties and circulation. Here, we review how recent advances in long-term monitoring programmes, process studies and newly developed numerical models have helped bridge these gaps and set future research challenges for the WAP system.

This article is part of the theme issue ‘The marine system of the West Antarctic Peninsula: status and strategy for progress in a region of rapid change’.

## 1. Introduction

The West Antarctic Peninsula (WAP) has undergone significant warming in the twentieth century, with observed changes reaching 3°C over the period 1955–2004 [1], as well as warming of the surface ocean of approximately 1°C [2] in the same period. The vast majority of glaciers along the WAP have also suffered significant ice loss [3,4], with a number of ice shelves collapsing in those decades. The WAP shelf is a highly productive ecosystem that sustains significant marine populations whose abundance and geographical distribution have been impacted by the changing ice conditions and warming atmospheric temperatures [5,6].

Understanding the current circulation and distribution of heat and salt on the WAP shelf is a fundamental step to understand future change, and how the coastal ocean will impact both the biological community and the regional climate. Much of our earlier understanding of the physical oceanography of the WAP shelf was based on traditional hydrographic surveys as part of either process studies or long-term monitoring programmes, including the Southern Ocean Global Ecosystem Dynamics programme (SO GLOBEC), the Palmer Long-Term Ecological Research programme (Palmer LTER) and the British Antarctic Survey’s efforts at Rothera Station [7–9]. Starting in the late 1990s and early 2000s, the surveys were augmented in critical ways with the deployment of moorings, of autonomous underwater vehicles, and the development of high-resolution regional atmospheric and ocean models able to resolve the relatively small dynamical scales that are typical of these high-latitude regions.

Here, we review our current understanding of the distribution and evolution of hydrographic properties as well as the subtidal circulation on the WAP shelf. We focus particularly on recent studies revealing the exchange mechanisms between the open ocean and the shelf, the fate of ocean intrusions on the coastal ocean, and critical aspects of the heat and salt budgets of the shelf. We also discuss briefly the long-term changes observed in this region, including the strong influence that climate modes such as El Niño–Southern Oscillation (ENSO) and the Southern Annular Mode (SAM) have on the inter-annual variability of the WAP. We discuss new questions revealed by these studies, and future challenges for the research community.

## 2. An overview of the hydrography and subtidal circulation on the West Antarctic Peninsula shelf

**Figure 1.**
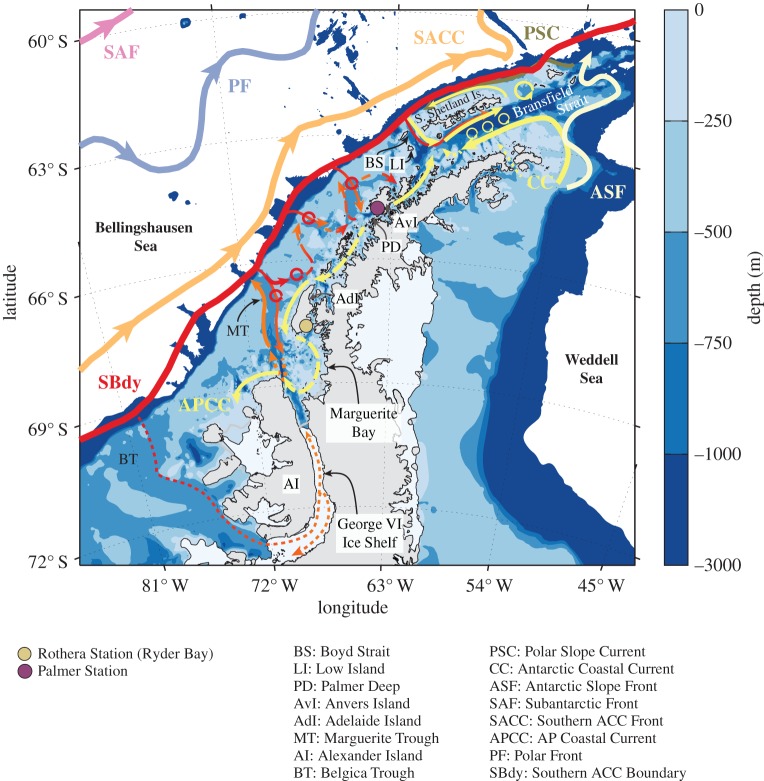
Overview of the circulation on the WAP shelf, based on multiple sources. Illustrated are the climatological location of the fronts and southern boundary of the ACC [10]. Solid lines indicate currents for which direct evidence exists, and dashed lines are suggested pathways. A version of this figure is available for use from https://doi.org/10.6084/m9.figshare.5954329.

The WAP continental shelf extends for some 1200 km from the southern boundary of Alexander Island to the tip of the Peninsula (figure 1). As is typical of polar, glacially carved continental shelves, it is characterized by steep, deep bathymetry.

The WAP can be broadly divided into two distinct regions: Bransfield Strait and the central WAP. Bransfield Strait forms an elongated basin oriented parallel to the coast, with depths exceeding 2000 m, and limited by the Peninsula to the southeast, by the South Shetland Islands to the northwest and by Boyd Strait to the south [11]. What we refer to as the central WAP shelf, between Low Island and Alexander Island, is characterized by typical depths of approximately 400 m and a number of troughs cutting across the shelf that deepen towards the coast. The largest is Marguerite Trough, which runs from the shelf break at about 66.5°S to George VI Ice Shelf in Marguerite Bay [12]. Our current understanding of the circulation on the WAP shelf, discussed below, is illustrated in figure 1, and reflects the competing influences of the Antarctic Circumpolar Current (ACC) and inflow from the Weddell Sea, run-off and glacier melt from the coast, and exchange with the atmosphere.

### (a) Bransfield Strait

The hydrography of Bransfield Strait is characterized by three distinct water masses. By volume, most of the Strait is occupied by a mixture of shelf and deep Weddell Sea waters with a characteristic temperature below 0°C and a minimum reaching less than −1.6°C in one of the three main basins in the Strait [11]. This Weddell-sourced water is found throughout the water column along the WAP mainland coast, including at the northern section of Gerlache Strait, and below approximately 100–150 m everywhere else away from the South Shetland Islands slope [13–16]. There, fresh (salinity less than 34.35) and warm (above −0.4°C) ‘Shelf Slope Water’ occupies the slope and extends about two-thirds of the Strait width towards the WAP mainland [17], forming a well-defined surface (approx. 100–150 m) front, sometimes called the Peninsula Front [18], that separates buoyant water from the cold, salty water of Weddell Sea origin. A second, sharp front, or Bransfield Front [19], of *O*(10 km) width is formed at depth along the slope of the Islands by the downward-sloping isopycnals of the buoyant Shelf Slope Water, and by a warm (0<*T*<1°C) and salty (*S*>34.50) water core found at varying depths ranging from 200 to 550 m. The latter is thought to be an intrusion of Circumpolar Deep Water (CDW) entering through Boyd Strait [13,19], while the shallow Shelf Slope Water appears to have contributions from both Boyd Strait inflow and water advected across the southern boundary from Gerlache Strait [20–23], and possibly across the gaps separating Bransfield Strait from the warmer, central WAP. It is also likely to be strongly influenced by meltwater from Shetland Islands glaciers [24], but this has not been fully quantified on the Strait.

The circulation in Bransfield Strait is dominated by a cyclonic gyre with a branch flowing southwest around the tip of the Peninsula [17,25], transporting the cold water from the Weddell Sea found shorewards of the Peninsula Front [18,26–28]. Along the northeast boundary (figure 1), the Bransfield Current resulting from the presence of the Bransfield Front carries the warmer, fresher Shelf Slope and CDW-sourced waters towards the northeast with velocities reaching 0.3–0.5 m s^−1^ [18,17,29]. Partial recirculation of the upper ocean flow around the South Shetland Islands has been observed, resulting in southward flow along the shelf slope northwest of the Islands [18,30]. Along that outer slope, at depths ranging from 200 to 600 m, shipboard and mooring observations also show a core of cold water originating from the Weddell Sea, the Polar Slope Current [13,31]. A more recent study using repeated hydrography along the Drake Passage shows that this export of Weddell Water along the north slope of Elephant Island is probably a result of wind-driven modulation on the Weddell Sea, with relatively warmer, saltier waters being exported to the WAP when wind stress becomes more cyclonic over the gyre [32]. The fate of this current has not been determined, but there is no evidence of it reaching Boyd Strait or the central WAP slope to the south.

The above structure in hydrographic properties and circulation results in stronger cross-shore than along-shore gradients in Bransfield Strait, which contrast to the central WAP discussed below. The modification and mixture of the three water masses in the Strait is not well understood, and neither is the connection between the southeastward-flowing current along the mainland with the Bransfield Current, or the fate of the latter as it reaches the northern tip of the Strait. Surveys do suggest the presence of isolated features with CDW water away from the Shetland Islands slope [14], which is consistent with an active eddy field observed between the Peninsula and Bransfield Fronts [18,25], probably a key mechanism for water exchange and modification within Bransfield Strait.

### (b) Central West Antarctic Peninsula

**Figure 2.**
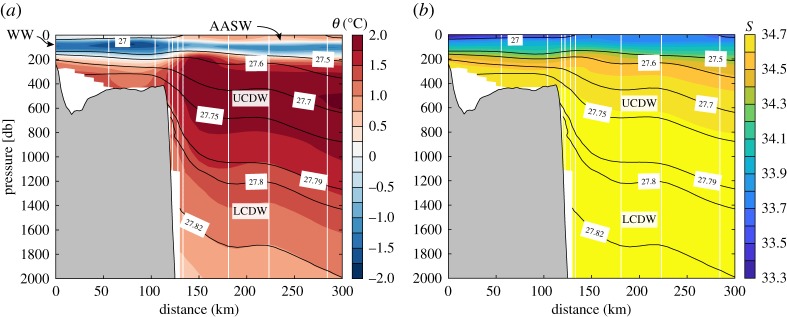
Cross-shelf hydrographic structure during the WOCE S04P cruise [33]. (*a*) The potential temperature (*θ*) and (*b*) the salinity. The black lines show selected isopycnals. Also labelled are the Antarctic Surface Water (AASW), Winter Water (WW), and Upper (UCDW) and Lower (LCDW) Circumpolar Deep Water (CDW).

**Figure 3.**
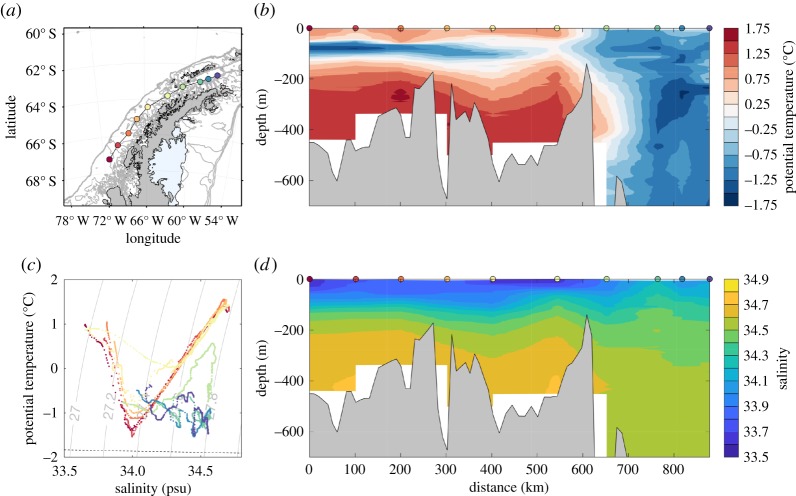
Hydrographic structure along the WAP shelf from selected stations collected in Austral summer 1998 (Dec 1997–Mar 1998). Panel (*c*) shows a *θ*–*S* diagram (colour coded by distance along the section), and (*d*) the corresponding vertical sections of potential temperature and salinity. Data obtained from the World Ocean Database 2013 [34].

In the central WAP, the hydrography is strongly influenced by air–sea exchange, the availability of CDW along the shelf slope and the melting of land ice. The surface layer is occupied by Antarctic Surface Water (AASW), a relatively cold and fresh water mass (figures 2 and 3). This layer undergoes significant changes throughout the year, as heat loss and formation of sea ice during the autumn and winter lead to temperatures close to the freezing point and salinities of about 33.5–34 in near-shore areas [35–37]. Warming and freshening of this layer follow in the spring and summer. This cycle results in a deep winter mixed layer that is not completely eroded in the spring and summer, thus forming the characteristic subsurface temperature minimum of Winter Water (figure 3). The deep hydrographic properties are dominated by a modified (colder and fresher) version of Upper Circumpolar Deep Water (UCDW), which is characterized by a temperature maximum, and Lower Circumpolar Deep Water (LCDW), characterized by colder temperatures than UCDW and a salinity maximum (figure 2). The latter is mostly found in Marguerite Trough [33] and other deep canyons on the shelf [38]. Intrusions of oceanic CDW supply heat and salt to the shelf (figure 2), and the modified CDW (mCDW) is also a primary source of inorganic macronutrients to coastal waters [39], although local sources (e.g. sediments, glacial meltwater) appear critical for the budget of micronutrients such as iron [40,41]. mCDW forms as a result of vertical heat loss to the surface layer, and mixing with cold, fresh water of glacial origin, although the heat balance, discussed in §5a, is still poorly constrained.

The near-surface circulation on the central WAP is characterized by flow towards the northeast along the outer shelf and slope, and towards the southwest, following the coast, near the shore (figure 1). The former is associated with the ACC, and the latter with the Antarctic Peninsula Coastal Current (APCC), a strong, narrow current forced by freshwater discharge and downwelling-favourable winds near the coast [42]. The ACC fronts (Subantarctic, Polar and Southern ACC) and southern boundary (SBdy in figure 1) flow roughly parallel to the shelf along the entire WAP, and the southernmost front and boundary are found closer to the continent than in most regions around Antarctica [10]. The APCC [42,43] is characterized by a strong cross-shelf density gradient with vertical and horizontal scales of 100–150 m and 20 km, respectively, and along-shore velocities of approximately 0.3 m s^−1^. Mooring data near Adelaide Island suggest a significant seasonal modulation of this current, with a diminished presence in the ice-covered months as run-off is reduced [42], although acoustic Doppler current profiler (ADCP) surveys (with measurements starting at 40 m depth) show a weak but sustained southwards flow south of Anvers Island in the winter [30]. Cyclonic, surface-intensified flow in Marguerite Bay and moving south along Alexander Island is consistent with buoyancy-driven, coastally trapped flow [30,42]. The along-shore extension (particularly in the northern WAP coast) and continuity of the APCC along the shelf are poorly constrained, but available evidence suggests that flow in Gerlache Strait is towards Bransfield Strait [25,44]. Moreover, mean wind forcing is upwelling-favourable along the coast in Bransfield Strait [45,46], which would tend to advect freshwater discharge offshore, implying the APCC has a northern limit near Anvers Island [30,42]. Dynamically, the APCC appears analogous to the Antarctic Coastal Current found elsewhere around the continent [27,47–49].

The shelf circulation below the pycnocline is strongly steered by the steep bathymetry. On the central WAP shelf, observations show that troughs cutting across the shelf are the main pathway for water to move towards the coast. Along Marguerite Trough, observations show a mean cyclonic circulation with inflow along the northwest bank and weaker outflow along the southeast bank [30,33]. A fraction of the inflow splits into a bathymetric fork to the northeast [38], with the rest appearing to continue to move shoreward into Marguerite Bay, consistent with regional modelling results [50,51]. Typical velocities in this layer are approximately 0.05 m *s*^−1^, and ocean eddies appear to follow these pathways as they enter the shelf (we explore this further in §4). Geostrophic maps from shelf-scale hydrographic surveys reveal broad cyclonic circulation patterns on the mid-shelf [52,53], although these surveys generally do not resolve the flow around steep, narrow bathymetric features. The surface circulation along the major troughs and banks on the shelf is less understood, as direct current observations in the top 50 m are difficult to collect, but drifter measurements [43] and modelling [50,54] suggest that the bathymetric steering of the flow often has a surface expression. Overall, the evidence points to deep troughs with strong cyclonic mean flows, contrasting with weaker circulation over submarine banks. A prominent example is the region off Adelaide Island (light blue areas in figure 1), where flow appears to be strongly steered around large shallow areas [52,53], probably forming significant retention areas with long residence times. At least four large banks are evident in the mid-shelf bathymetry between Adelaide and Anvers Islands, suggesting that multiple such retention areas are likely to exist, although this requires further study.

## 3. Along-shore property gradients and glacier retreat

The variability of the bathymetry and the relative influence of ACC- and Weddell-sourced waters in Bransfield Strait and the central WAP lead to a strong along-shore gradient in hydrographic properties. In Bransfield Strait, CDW intrusions are observed but are relatively cold, and are steered northeastwards along the South Shetlands Islands, with colder Weddell Waters dominating the hydrographic structure along the coast of the WAP mainland. By contrast, warmer CDW intrusions find a direct pathway across the shelf through several deep troughs in the central WAP, resulting in relatively warm near-shore conditions along that southern region.

The magnitude and forcing mechanisms of along-shore exchange between Bransfield Strait and the central WAP shelf are not well understood. The strongly sloping bathymetry in Bransfield Strait, and the relatively shallow bathymetry around the islands at the boundary with the central WAP, probably inhibit exchange between these two regions (figure 3). The wind forcing also shows a strong divergence at this boundary, with climatological means generally showing northward (upwelling-favourable) winds in Bransfield Strait and southward (downwelling-favourable) winds in the central WAP [45,46]. A fraction of the flow coming from the Weddell Sea enters Gerlache Strait, but several studies have shown that, at least at the surface, most of it recirculates back and across towards the South Shetland Islands [20,22,25,30].

The resulting hydrography shows a sharp property gradient in the region separating the waters of Bransfield from the central WAP (figure 3), particularly near the WAP mainland. Studies of the evolution of glacier retreat along the Peninsula have recently revealed a strong modulation by this along-shore temperature gradient, with the colder waters in Bransfield Strait leading to slower rates of retreat than in the central WAP [55]. The warming appears to have been more vigorous in the CDW-dominated central WAP, suggesting a divergence in the evolution of properties across this boundary. Understanding the dynamics and magnitude of heat supply to these regions and the property exchange along the shelf is critical for projections of glacier retreat in the Peninsula.

## 4. Ocean–shelf exchange processes and supply of heat to the shelf

The processes controlling the inflow of CDW onto the central WAP shelf, and its transport and modification as it moves across it, have been the subject of several observational and modelling studies. Analyses of hydrographic data from early broad-scale cruises suggested the inflow of CDW occurs as oscillations of the ACC flood broad regions of the shelf and identified the rugged topography as preferred pathways for the circulation [52,56]. Time series from moored arrays collected during the SO GLOBEC in the early part of this century [7,57], the International Polar Year’s Synoptic Slope-Shelf Interaction (IPY SASSI) and Palmer LTER programmes confirmed that bathymetric features like Marguerite Trough (figure 1) are key pathways for shelf–ocean exchange, but also revealed that CDW is delivered to the shelf by ocean eddies with relatively small horizontal scales (approx. 5 km) and vertical scales of 100–200 m [33,38]. An examination of alternative intrusion mechanisms, including upwelling of deep water at the shelf break [39] using the LTER/IPY SASSI time series, concluded that eddies are probably the most important delivery mechanism of upper CDW to the shelf [38].

Eddies are detected in the moorings at weekly time scales, have a generally weak horizontal circulation and appear to be carried onshore embedded in a cross-shelf flow that is strongly steered by bathymetry, i.e. along large submarine features like Marguerite Trough [33]. However, characterizing the shelf-wide distribution of eddies using a small set of moorings or coarse-scale hydrographic surveys can be difficult given their small scales and synoptic-scale frequency. Increased deployment of autonomous underwater vehicles (AUVs) on this shelf has helped to bridge this critical observational gap, with a recent study characterizing the eddy ‘population’ over the shelf and providing a unique view of the synoptic spatial structure of the eddies. Based on late spring and summer AUV deployments over three consecutive years (2010–2013), the data revealed more than 30 eddies with widths of *O*(10 km) and vertical scales of 125 m being advected onshore preferentially at Marguerite Trough and another trough farther north in the central WAP [58].

Observational evidence for the dynamical origins of ACC inflow across the shelf break by eddies or other processes is still lacking, but numerical modelling studies have shed light on potential mechanisms. An early, non-eddy-resolving regional model showed that flow–topography interactions at bends of the shelf break, modulated by synoptic-scale along-slope winds, result in large inflow into Marguerite Trough [54]. Recently, the horizontal model resolution has been increased to 1.5 km, revealing enhanced eddy-scale fluxes in Marguerite Trough and other similar bathymetric features of the shelf [59]. Idealized numerical models of flow–topography interactions have shown that Rossby waves propagating along the shelf break and interacting with submarine troughs generate shoreward-propagating eddies with scales consistent with observations in Marguerite Trough [60]. They also show that the interaction of an ACC-like flow over topography results in a sharp potential vorticity front that, perturbed by the ACC, generates topographic waves and a clockwise circulation in an idealized, flat-bottomed area representing Marguerite Trough [61]. This cyclonic circulation and the formation of topographic waves in the channel are consistent with observations of flow in Marguerite Trough [33,62]. Finally, an idealized model of a jet over bathymetry slightly larger than the eddy scale, but without including cross-shelf topography, found the development of intermittent baroclinic instabilities that were correlated to enhanced cross-slope exchange [63].

Overall, both observational and modelling studies support the importance of ocean eddies in the transport of CDW water from the ACC to the WAP shelf. These findings are consistent with studies of eddy-modulated transport elsewhere in Antarctica [64,65]. A number of modelling studies have proposed plausible mechanisms for the formation of eddies along the shelf break as the ACC, possibly modulated by wind forcing, interacts with the slope. Observations along the slope and off the shelf are critical to elucidate the mechanisms originating the eddies and forcing them onshore.

## 5. Budgets of heat and salt on the West Antarctic Peninsula shelf

### (a) The heat budget and ventilation of UCDW

**Figure 4.**
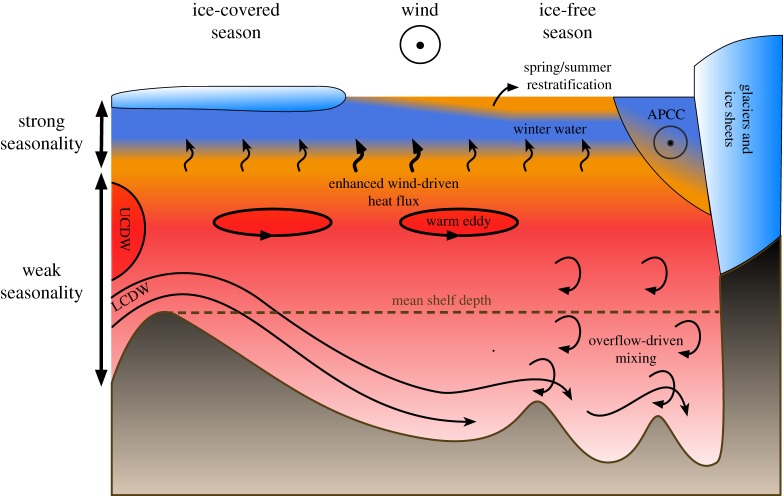
Key features of the circulation and heat budget on the central WAP shelf. The surface layer is forced by (on average) northerly winds, and undergoes strong seasonal change. Heat flux from the lower layer is significant year-round, but it is enhanced after the seasonal ice breaks up, but before the surface layer restratifies. Heat is supplied at depth from the ACC by mean flows, eddies and deep overflows carrying CDW. Mixing of this inflow also occurs at topographic features. Along the coast, a southward buoyancy-driven current (APCC) transports fresh, cold water. A version of this figure is available for use from https://doi.org/10.6084/m9.figshare.5954329.

The heat budget of the central WAP shelf has been approximated as two-dimensional (figure 4), with inflow of CDW (both as UCDW and LCDW branches) supplying heat and salt to the shelf. The deep water becomes cooler and fresher as vertical fluxes through the pycnocline balance the net cooling to the atmosphere as well as precipitation [66]. This conceptual model can be made more realistic by including a region near the coast where melting glaciers and ice shelves [55,67] are a source of cold and fresh water at varying depths, including below the pycnocline [68,69]. A recent estimation [58] of the magnitude of the horizontal and vertical components of the heat budget suggests that approximately two-thirds of the heat advected across the shelf break is fluxed upwards through the pycnocline, with the remainder delivered to the near-shore region. A significant fraction (up to 53%) of the lateral heat flux into the central WAP shelf can be attributed to eddies entering through Marguerite Trough [58].

The estimations above are critically dependent on educated guesses of the magnitude of the horizontal and vertical diffusivities that modulate the decay of the eddies as they move across the shelf. More generally, understanding the processes that control the modification and ventilation of the deep water, and how they vary in time and space, is a key step for closing the property budgets. Several recent studies shed light on these mechanisms, and highlight strong temporal and spatial variability of the dominant mixing processes on the shelf.

Detailed hydrographic observations collected in several deep troughs in the WAP, including Marguerite Trough and Palmer Deep [70], show shoreward flow of mCDW encountering steep topographic obstacles. This process results in overflow of water above the blocking depth to the next deep depression, a process accompanied by strong mixing (figure 4). The mixing erodes the mid-depth temperature maximum associated with mCDW that is typical of profiles on the mid and outer shelf. The deep water initially warms as heat is fluxed downwards from the mid-depth maximum, but can eventually cool as entrainment with shallower water pulls colder, fresher water from the thermocline. This study [70] highlights the importance of flow–topography interactions on the shelf in determining the deep water properties, particularly in near-shore regions where the inflow from the ACC has suffered the most significant modification through several deep ridges (figure 4). The variability of along-shore properties is probably strongly modulated by the pathway of the flow from the shelf break, challenging the ability of a two-dimensional model of the shelf to explain hydrographic variability near the shore.

The upward mixing of the heat and salt in the mCDW to the surface is a key step to close the property budgets. Early attempts at quantifying the intensity of the mixing through the pycnocline [71] resulted in estimated vertical diffusivities less than or equal to 10^−5^, corresponding to a heat flux to the surface of less than 2 W m^−2^ driven mostly by wind-forced shear instability. Although on the WAP shelf cold and fresh water overlies warm and salty water, double diffusive fluxes were deemed too small to make a significant contribution [71]. In near-shore sites, internal tides can contribute to vertical mixing [72]. Examining the potential sources for vertical heat fluxes, strong wind events resulting in enhanced vertical mixing or upwelling of UCDW along the coast were suggested [71].

A recent observational study made significant progress in understanding the sources and temporal variability of mixing on the shelf [73]. Using a 2.5 year-long time series of ocean velocity profiles, hydrographic profiles and wind velocity from Ryder Bay (figure 1), wind-driven currents are shown to be a key source for mixing, with the time variability and intensity of this process being heavily modulated by the timing of ice retreat. During fast-ice-free periods, turbulent dissipation is at a minimum and appears driven by diurnal-scale tides. When fast ice is absent, anticlockwise, broadband near-inertial energy that is well correlated with local wind forcing dominates instead. Turbulent dissipation rates increased roughly by an order of magnitude between ice-covered to ice-free periods in 2006. Pycnocline stratification, however, is largest during the ice-free season, and thus the resulting time-averaged heat fluxes (approx. 1 W m^−2^) did not differ significantly between the two seasons. Critically, the maximum heat fluxes—exceeding approximately 1.5 W m^−2^—were typically found early in the ice-free season, as the wind can first act on the water column directly, but before the stratification (driven mostly by salinity changes) increases. Overall, the results suggest that both the seasonal changes in ice cover and stratification and the synoptic-scale variability of the wind play an important role in modulating the upward flux of UCDW into the surface mixed layer. It also highlights the importance of long-term time-series studies—spanning several seasons—that can capture this variability.

### (b) The surface freshwater budget

Assessments of the spatially and temporally varying salinity across the WAP shelf reveal the changing net freshwater budget and information on its spatial structure. However, they do not inform *a priori* on the source of the fresh water being delivered to shelf waters. Additional tracers are useful in this context, including *δ*^18^O, the standardized ratio of stable isotopes of oxygen (H_2_^18^O and H_2_^16^O) in seawater. The utility of this tracer at the WAP derives from its different values in the different forms of fresh water being injected to the ocean: fresh water derived from sea-ice melt is relatively isotopically heavy (comparatively enriched in the heavier H_2_^18^O molecule), because the isotopic composition of the sea ice is similar to that of the seawater from which it originally formed. By contrast, the injection of meteoric water (i.e. the combination of glacial discharge and precipitation) adds much isotopically lighter water to the ocean, because evaporation in the low latitudes and poleward transport of atmospheric water vapour lead to very low values of *δ*^18^O in Antarctic precipitation and glacial ice. Consequently, when measured from the same seawater samples from which salinity is determined, *δ*^18^O provides additional insight into the origin of the fresh water that the sample contains [74].

**Figure 5.**
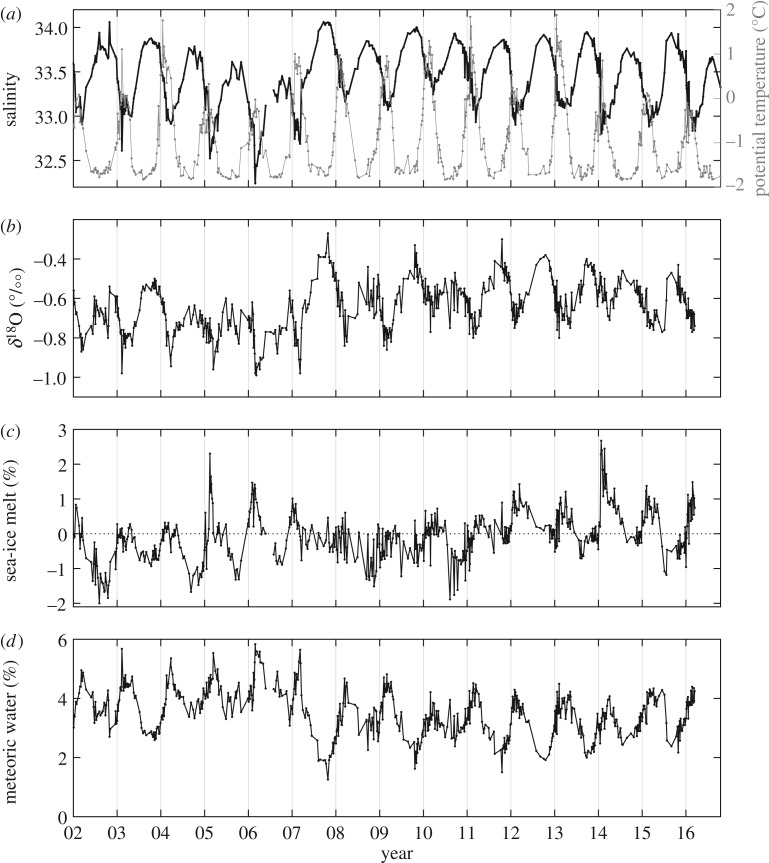
Time series of salinity and potential temperature (*a*), δ^18^O (*b*), sea-ice melt percentage (*c*) and meteoric water percentage (*d*) from the Rothera Time Series [79].

Stable isotope tracers have been used in a number of studies at the WAP, with particularly detailed information obtained from the ocean close to Rothera Research Station (figure 1). The Rothera Time Series (RaTS), located adjacent to northern Marguerite Bay, has provided almost uninterrupted quasi-weekly sampling of the ocean year-round since it started in 1997 [75,76]. Sampling for oxygen isotopes at RaTS commenced in 2002, and a sequence of papers have investigated the seasonal, inter-annual and decadal-scale signals in the freshwater budget using these data [37,77–79]. Figure 5 shows the most recently presented RaTS salinity and *δ*^18^O data from 15 m depth, along with sea-ice melt and meteoric water derived using a simple three-endmember mass balance [80].

This unique time series reveals that meteoric water at 15 m typically varies seasonally by around 2%, generally equal to or larger than the corresponding signal in sea-ice melt. This is caused by a combination of processes, including seasonality in the run-off of glacier melt, and snow accumulation on sea ice which breaks up and melts each year. Sea-ice melt, in turn, varies between around −2% and +2% over the course of the series, with negative values indicating a net sea-ice production prior to the time of sampling.

Inter-annual changes in both meteoric water and sea-ice melt are related to coupled modes of climate variability, in particular the SAM and ENSO. These modes strongly influence the wind fields, atmospheric temperature and precipitation at the WAP, which in turn impact sea-ice formation, advection and melt, and also the injection of meteoric water to the ocean. A recent modelling investigation of the dynamics whereby SAM and ENSO control the freshwater inputs at the WAP, with emphasis on the meteoric components [81], showed that La Niña events during high SAM periods result in higher snowfall rates in Marguerite Bay, while the same SAM phase during El Niño events results in warmer air advection over the WAP, reduced snowfall and increased rain.

A particularly strong determinant on both meteoric water and sea-ice concentrations at RaTS is the mixed layer depth (MLD) at the time of sampling. MLD changes seasonally, inter-annually and decadally, with particularly deep winter mixed layers observed during extremes of ENSO and/or SAM. Deeper mixed layers have the consequence of reducing the concentrations of surface-injected fresh water at a fixed sampling level, by mixing that fresh water over a greater depth interval. The long-term decline in meteoric water apparent in figure 5 is predominantly a consequence of MLD increasing over this time period, rather than a reduction in meteoric water input to the ocean.

RaTS provides unprecedented information on the temporally varying freshwater composition at the WAP, but (like all time series) is representative of a single location. To broaden the spatial knowledge of the sources of freshwater input to the WAP ocean, *δ*^18^O sampling commenced in 2011 as part of the Palmer LTER programme [82]. These *δ*^18^O datasets, collected each January, reveal that meteoric water is typically elevated inshore, due to the proximity of the glaciers and orographic effects on precipitation, with values around 4–5% along the inner shelf region, falling to around 2% near the shelf break [79]. This spatial pattern is not constant over time, however; for example, the inshore enhancement of meteoric water in January 2013 was greatly reduced due to anomalously low precipitation in the last quarter of 2012. Of particular note also is that sea-ice motion across the WAP shelf can be traced in these data: some cruise datasets (e.g. January 2011) show isotope-derived sea-ice production in the northern part of the sampling grid and melt in the southern part, indicative of a net southward ice motion induced by northerly winds. By contrast, other years show no such spatial structuring in the isotope-derived production and melt, corresponding with more southerly winds restricting southward ice motion during the melt season. The data from January 2014 show a particularly pronounced example of this, and are coincident with the maximum in sea-ice melt ever observed in the RaTs site time series.

## 6. Assessing long-term change in the West Antarctic Peninsula shelf

Observational studies show significant long-term trends in key ocean properties. The warming of the near-bottom waters of the WAP has been documented in multi-decadal time scales [83], and is associated with both warming of the CDW and rising of its core within the ACC. A study using data from 1955–1994 showed warming of the upper layers of the shelf exceeding 1°C, and accompanied by salinification, both of which were attributed to the warming of the atmosphere and reduced ice production [2]. Consistently with those results, an evaluation of the data from the first 12 years (1993–2004) of the LTER summer cruises showed the average heat content has increased, which was partially attributed to enhanced heat flux from the ACC and warming of CDW at the slope [53]. However, the LTER data also revealed a decreasing trend (0.6 W m^−2^ *yr*^−1^) in the heat flux from the surface ocean to the atmosphere, suggesting the heat build-up in the water column could be partially caused by a reduced ability of the deep UCDW to be ventilated upwards [53].

The above trends apply to the central WAP shelf, which is the region most directly impacted by the ACC. The LTER grid that is regularly sampled does not include the Bransfield Strait region. Although not addressed explicitly, the studies mentioned above [2,83] appear to show weak to negative temperature trends in the Bransfield Strait region. This probably reflects the influence of the Weddell Sea, where a deepening of the CDW core has been observed [83]. These differences in along-shore structure are also consistent with observations of substantial reductions of the chlorophyll concentration in Bransfield Strait and significant increases in the central WAP [5], as well as differential rates of glacier retreat along the shelf [3,55]. This warming was accompanied by significant changes in ice coverage. Satellite-derived observations of ice show a reduction of the ice-covered season length by about 85 days from 1979 to 2004 [84]. This trend was associated with a tendency towards a more positive SAM and several contemporaneous La Niña events during the 1990s, which led to strengthened northerly winds along the WAP in autumn and spring, and consequently earlier sea-ice retreat and later sea-ice advance.

The long-term warming trend of the central WAP shelf has been associated with a well-documented, contemporaneous rise in atmospheric temperatures, but a recent study shows a reversal of this trend starting in the late 1990s [85]. This dramatic change was attributed to more frequent cyclonic conditions in the lower atmosphere of the Weddell Sea. In turn, this results in more frequent cold events and advection of sea ice into the WAP region. Critically, the summer LTER data show that the ocean heat content continued to increase throughout at least the early period (ending in 2004) [53] after the atmosphere shifted to cooling [85]. Extending these analyses to a longer period after the transition might well reveal a reversal in ocean property trends, particularly as winds strongly modulate vertical mixing, CDW depth and air–sea exchange fluxes [86,87]. However, given the uncertainties in our understanding of the processes forcing CDW supply to the shelf, this remains an open question.

## 7. Summary and future challenges

Both modelling and observational efforts are required to better understand the supply of CDW to the shelf. A consistent body of research now shows that small ocean eddies are critical to explain the supply of UCDW [33,38,58,59], while LCDW probably results from a more steady overflow into deep troughs on the shelf [33]. Key to these advances are long-term mooring arrays and AUV surveys, which provide the required eddy-resolving resolution in time and space. There have been significant modelling efforts to understand the dynamical origin of these eddies, but additional observations from the continental slope will be critical to determine which single or combination of mechanisms put forward in those studies is responsible for the CDW supply to the shelf.

The inflow of ocean heat to the deep layers of the central WAP is compensated by a combination of heat loss to the surface layer (and, from there, to ice formation and the atmosphere), and to the near-shore regions where warm ocean waters modulate glacier melt and retreat. Recent process studies highlight the importance of small-scale topography and seasonal transitions in sea ice and stratification on the variability and magnitude of the mixing of mCDW on the shelf, and its ventilation through the pycnocline. There remains, however, a lack of reliable measurements of diapycnal diffusivity based on microstructure sensors, using either shipboard profilers or those mounted on AUVs or moorings. These observations are key for advancing our understanding and quantification of these mixing processes. Also, a natural next step is to evaluate how well represented these processes are in newly developed, eddy-resolving regional numerical models, and their impact on the heat and salt budgets of the shelf. Finally, the impact that distinct mechanisms of delivery of heat, salt and nutrients (e.g. strong mean flows in large troughs, eddies carrying UCDW, overflows of LCDW) have on biological communities and biogeochemical cycles on the WAP is still poorly understood, despite their importance.

Sustained observations from the surface across the pycnocline and mixed layer and extending throughout the winter remain a significant observational challenge in polar environments such as the WAP. Year-round, long-term efforts such as RaTS provide unique and novel insights into the surface properties and the freshwater budget. On the wider shelf, near-surface observations remain scarce, and the surface circulation is generally poorly constrained. Yet, they remain a critical link to a better understanding of the ventilation of CDW, the advection of fresh water and sea ice, and the influence of near-shore coastal processes on shelf dynamics. At seasonal, inter-annual and longer time scales, these observations are needed to determine the distinct influence of local and remote atmospheric forcing on the WAP oceanography, particularly as tropical teleconnections and regional modes of variability show a competing influence on the shelf throughout the year.

## Data accessibility

This article has no additional data.
